# Alternative Donor Transplantation for Acute Myeloid Leukemia

**DOI:** 10.3390/jcm4061240

**Published:** 2015-06-09

**Authors:** Nelli Bejanyan, Housam Haddad, Claudio Brunstein

**Affiliations:** 1 Division of Hematology, Oncology and Transplantation, University of Minnesota, 420 Delaware Street SE, Mayo Mail Code 480, Minneapolis, MN 55455, USA; E-Mail: bruns072@umn.edu; 2 Hematology and Oncology Department, Staten Island University Hospital, 475 Seaview Ave, Staten Island, NY 10305, USA; E-Mail: husamhaddad@yahoo.com

**Keywords:** AML, alternative donor, UCB, Haploidentical, Transplantation

## Abstract

Allogeneic hematopoietic cell transplantation (allo-HCT) is a potentially curative therapy for adult patients with acute myeloid leukemia (AML), but its use for consolidation therapy after first remission with induction chemotherapy used to be limited to younger patients and those with suitable donors. The median age of AML diagnosis is in the late 60s. With the introduction of reduced-intensity conditioning (RIC), many older adults are now eligible to receive allo-HCT, including those who are medically less fit to receive myeloablative conditioning. Furthermore, AML patients commonly have no human leukocyte antigen (HLA)-identical or medically suitable sibling donor available to proceed with allo-HCT. Technical advances in donor matching, suppression of alloreactivity, and supportive care have made it possible to use alternative donors, such as unrelated umbilical cord blood (UCB) and partially HLA-matched related (haploidentical) donors. Outcomes after alternative donor allo-HCT are now approaching the outcomes observed for conventional allo-HCT with matched related and unrelated donors. Thus, with both UCB and haploidentical donors available, lack of donor should rarely be a limiting factor in offering an allo-HCT to adults with AML.

## 1. Introduction

Allogeneic hematopoietic cell transplantation (HCT) is widely used as a curative therapy for acute myeloid leukemia (AML). The use of reduced intensity conditioning (RIC) extended eligibility of HCT to older adults and those with comorbid conditions [1,2,3,4]. However, donor availability for many adults with AML still remains a significant challenge because HLA-identical matched sibling donors (MSD) or adult unrelated donors (MUD) are available for only about 60% of patients [5]. As the age cutoff for RIC HCT eligibility has increased, there has been a critical need for alternative donors for those who may not have a suitable HLA-matched MSD or MUD donor. Moreover, because high-risk AML is more common among the elderly, the time it takes to secure a MUD [1,2] increases the risk of leukemia relapse in this group who need to proceed to HCT promptly. Thus, in recent years unrelated umbilical cord blood (UCB) or haploidentical grafts have been studied as alternative donor types for adults with acute leukemia [1,2,3,4,6,7,8,9,10]. In this manuscript, we review the outcomes of HCT with these two alternative donor types in the management of adults with AML.

## 2. Umbilical Cord Blood Transplantation

UCB has been increasingly used for the past two decades as an alternative donor type given its rapid availability, less restrictive HLA-selection criteria, no donor risk, and relative low risk of graft-*versus*-host disease (GVHD). The introduction of double UCB (dUCB) transplantation extended access of UCB to most adults with hematological malignancies, including acute leukemia. However, barriers to UCB transplantation include limited stem cell content, delayed engraftment accompanied by increased risks of infectious complications, and cost. Several strategies have been used recently to improve the clinical outcome of UCB transplantation, such as achieving faster engraftment and further minimizing the incidence of GVHD without compromising immune reconstitution. Such promising strategies include the expansion of cord blood progenitor cells by various techniques [11,12,13], intra-bone marrow injection of cord blood cells [14,15,16] to improve hematopoietic engraftment, and use of Tregs to reduce risk of GVHD after UCB transplantation [17].

## 3. Myeloablative Single UCB Transplantation

Initial reports on the use of UCB as a donor type for hematopoietic cell transplantation (HCT) were based on the use of single UCB grafts and largely limited to pediatric patients [18,19,20,21,22,23,24]. At that time, the largest barrier to the use of UCB in adults was the weight of these patients relative to the limited cell dose available in individual UCB units. The first study to focus on UCB transplantation of adults reported on 68 patients [25] who were heavily pretreated and had high-risk hematological malignancies, 19 of whom had AML. The nucleated cell dose used in that study was inadequate considering today’s standard; however, the cell dose used was based on available data largely from pediatric studies. Not unexpectedly, hematopoietic recovery was slow and treatment-related mortality (TRM) was high (47% at three months), resulting in poor leukemia-free survival (LFS) (26% at 40 months). That study found better outcomes among patients who received higher total nucleated (TNC) (≥2.4 × 10^7^/kg) and higher infused CD34+ (≥1.2 × 10^5^/kg) cell doses. In addition, the adult cohort of the Cord Blood Transplantation (COBLT) prospective study observed poor outcomes, mainly owing to inadequate TNC dose and the use of UCB as a “last resort” effort for very high-risk patients [26]. Despite limitations, these studies demonstrated the feasibility of UCB allografting in adults and set the stage for future studies seeking to improve outcomes among UCB recipients.

While many centers started using double UCB transplantation (reviewed below) to achieve an adequate cell dose for adult patients who are heavier than pediatric patients, many centers remained interested in single UCB transplantation either per institutional or country policy. Moreover, in recent years the availability of a larger inventory of UCB units has further improved the chances of finding adequate single-UCB unit grafts for adult transplantation. Takahashi *et al.* identified that, despite a delay in hematopoietic recovery, UCBT was associated with a markedly lower rate of chronic GVHD as compared to MRD transplantation [27]. More recently, the Valencia group reported their experience with single UCB transplantation after myeloablative conditioning in adults with higher-risk AML [28,29]. They used busulfan (BU)-based chemotherapy as a conditioning regimen and a UCB graft selection strategy based on improved cord blood banking standards that take into account the CD34+ cell count at the time of cryopreservation. They observed that median neutrophil engraftment occurred at 19 to 20 days and that disease-free survival (DFS) at five years was approximately 40%. Notably, patients with AML in first complete remission (CR1) who received a TNC dose ≥2 × 10^7^/kg had a DFS at four years of 75%. In the most recent report, they also showed that patients receiving less well-HLA-matched UCB grafts had a lower risk of relapse and superior LFS [30]. Another important advance in single UCB transplantation is the strategy of delivering the graft, often with a TNC dose below current standards, directly into the bone marrow (known as intra-bone marrow infusion, IBMI) [14].

## 4. Myeloablative Double UCB Transplantation

The University of Minnesota pioneered the use of double UBC transplantation, which was developed to overcome the limitation of infused cell dose in adults and serve as a platform for graft manipulations [19]. The first series of double UCB recipients included 23 adult patients with high-risk leukemia using a myeloablative conditioning regimen consisting of cyclophosphamide (Cy; 120 mg/kg), fludarabine (Flu; 75 mg/m^2^), and total body irradiation (TBI; 1320cGy) [31]. In this case series, double UCB transplantation led to improvements in median infused TNC dose (3.5 × 10^7^/kg), sustained neutrophil engraftment (median of 23 days), and DFS at one year (57%). This success was in part due to no graft failure events and a low rate of TRM (22%). While the risk of acute GVHD (65%) with double UCB was higher than that seen in single UCB transplantation, it was largely due to an increase in grade II acute GVHD. The risk of chronic GVHD, however, was still low. Thus, the strategy of double UCB unit infusion became widely used, with other transplant centers investigating different preparative regimens and post-transplant immunosuppression [32,33,34,35]. While some preparative regimens were found not to support this treatment platform [32], variations of the myeloablative Cy/Flu/TBI regimen resulted in similar clinical outcomes, allowing many transplant centers worldwide to utilize double UCB transplantation for many adults with AML who required myeloablative conditioning [4,31,34,35]. The dissemination of this strategy, at least in part, was due to its simplicity, as any center technically able to thaw and infuse single UCB grafts was able to take advantage of the double UCB platform to extend transplantation to larger patients.

## 5. UCB Transplantation with Reduced Intensity Conditioning Regimen

The introduction of RIC extended the use of allogeneic HCT to older, less clinically fit, and extensively pre-treated patients, such as those who had previous autologous transplant. This transplant approach is particularly important for patients with AML as it typically presents in their late 60s, an age in which the morbidity and mortality of a conventional myeloablative regimen would be excessive. Furthermore, older patients may lack an HLA-matched sibling donor who is healthy enough to donate, making alternative donor transplantation necessary for this group of patients. Moreover, high-risk AML subtypes, such as secondary AML, for which allogeneic transplantation is the only potentially curative treatment option, is more frequent among older patients as well [36,37]; for such patients, long-term survival with chemotherapy alone is generally poor [37]. Thus, the advantage of RIC HCT using UCB for older patients is its rapid availability, which helps to avoid further delay in proceeding with a potentially curative HCT.

One of the most commonly used platforms for RIC HCT using UCB is the one developed at University of Minnesota that consists of Cy 50 mg/m^2^, Flu 200 mg/m^2^ divided in five days, and TBI 200 cGy with cyclosporine A (CSA) and mycophenolate mofetil (MMF) for immune suppression [38,39,40,41,42,43]. Variations on this platform, which have led to promising results, include the use of treosulfan by the Seattle group [44] and thiotepa (Thio) by the MSKCC group [45]. The backbone of the conditioning platform (Cy 50 mg/m^2^, Flu 200 mg/m^2^, TBI-200) has been used to support single and double UCB transplantation according to various institutional practice criteria and has been shown to result in sustained donor engraftment in >90% of recipients, TRM between 20%–30%, and long-term DFS in 25%–50% of patients depending on disease stage and the presence of co-morbid conditions prior to transplantation [4]. The Boston group has also reported on an equally promising regimen that includes the combination of Flu, melphalan (Mel), and rabbit ATG [3,46], and when sirolimus/tacrolimus was used for immune suppression, a very low risk of GVHD was observed [46]. In addition, these overall encouraging results of RIC UCB HCT have been recently reproduced by two multicenter phase II studies by the Blood and Marrow Transplant Clinical Trials Network (BMT-CTN) [7] and the Societé Française de Greffe de Moelle Osseuse et Therapie Cellulaire and Eurocord [47,48,49].

## 6. Double *versus* Single UCB Graft

Several reports demonstrate that clinical outcomes among recipients of double and single UCB grafts are similar [42,43,47,50]. A recent registry study in patients with acute leukemia (*n* = 409; 285 AML) compared adults who received an adequate single UCB graft defined as a TNC dose of ≥ 2.5 × 10^7^/kg *vs.* double UCB grafts [42]. This study showed no difference in outcomes between one or two unit grafts. However, this conclusion has not been uniformly supported for relapse and acute GVHD. In some studies, a higher rate of AML relapse with single UCB transplantation was reported [43,51,52], but in other studies, no such association between number of infused UCB units and relapse or long-term treatment failure was observed [42,47,50,53,54]. In addition, although a higher risk of acute GVHD with double UCB was reported in one study [55], no difference in the risk of acute GVHD between single and double UCB transplantation was seen in other studies [53,54]. This discrepancy may in part be explained by differences in the patient population and use of ATG as part of the conditioning regimen. Additional evidence supporting the comparability of single and double UCB transplantation includes a recently reported prospective, multicenter, randomized, phase III study comparing single *vs.* double UCB grafts in children [56]. Outcomes were similar between the two groups. This study demonstrated that if a suitable single unit is available, there is no advantage in using a double UCB graft. However, an adequately dosed single-unit UCB graft cannot be frequently found for adults. Most double-unit UCB recipients would not have been eligible for UCB transplantation if an adequately dosed graft could not be generated with two UCB units. Thus, in adults who rarely have an adequate single UCB unit that meets the minimum cell dose criteria, a double UCB graft remains the standard of care.

## 7. UCB Grafts *versus* Other Donor Sources

Many retrospective studies have compared the outcomes of UCB to those of matched and mismatched URD in various settings including single [57] and double UCB [58] allografting and myeloablative [27,57,58,59,60,61] and RIC regimens [39,62,63,64,65,66,67] (Table 1). Notably, most studies have shown similar long-term outcomes between UCB and URD [27,58,61,67,68]. These studies demonstrated that UCB recipients had slower hematopoietic recovery [27,57,58,59,60,66], higher TRM [59,64,65,66,69], and often lower rates of acute grade II-IV and chronic GVHD than URD recipients [39,58,60]. Atsuta *et al*. reported higher TRM (30% *vs.* 19%, *p* = 0.004) and inferior survival (HR = 1.5; 95% CI 1.0–2.0; *p* = 0.028) among UCB recipients than URD recipients. As compared to other reports, this discrepancy can be explained in part by the majority of UCB recipients receiving a median TNC dose of 2.5 × 10^7^/kg, an inadequately dosed UCB graft by today’s standards [69]. In summary, the delay in hematological recovery and early TRM among UCB recipients observed in most studies was at least in part offset by lower risk of chronic GVHD and its complications and, in some series, remarkably lower risk of relapse, resulting in survival rates similar to other donor types. Novel strategies to improve the safety and efficacy of UCB transplantation are under way and have been reviewed elsewhere [70].

**Table 1:** Comparative allo-HCT studies of UCB with other donor types for acute leukemia in adults.

Reference	Malignancy	Donor Type	No of Patients	Median Age (range)	Median Time to ANC ≥500/µL	Median Time to Platelet >20 × 10^9^/L	aGVHD (II-IV) CI (%)	cGVHD CI (%)	TRM	Relapse	DFS
**Myeloablative conditioning**											
Laughlin 2004	Hematologic Malignancy 200 AML	UCB	150	(16–60)	27 days	60 days	0.81	1.62	1.89	0.73	1.48
		URD (BM)	367	(16–60)	20 days	29 days	0.66	1.12	0.99	0.85	0.94
							*p* = 0.17	*p* = 0.02	*p* < 0.001	*p* = 0.16	*p* = 0.001
		MM URD (BM)	83	(16–60)	18 days	29 days	1.0	1.0	1.0	1.0	1.0
					*p* < 0.001	*p* < 0.001	*p* = 0.04	*p* = 0.69	*p* = 0.96	*p* = 0.65	*p* = 0.69
Rocha 2004	Hematologic Malignancy 362 AML	UCB	94	25	26 days	--	0.57	0.64	1.13	1.02	0.95
				(15–55)							
		URD BM	584	32	19 days	--	1.0	1.0	1.0	1.0	1.0
				(15–59)	*p* = 0.001		*p* = 0.01	*p* = 0.11	*p* = 0.50	*p* = 0.93	*p* = 0.70
Takahashi 2004	Hematologic Malignancy 54 AML	UCB	68	36	22 days	40 days	0.61	0.60	0.32	0.75	0.27
				(16–53)							
		URD BM	45	26	18 days	22.5 days	1.0	1.0	1.0	1.0	1.0
				(16–50)	*p* = 0.01	*p* < 0.01	*p* = 0.05	*p* = 0.18	*p* = 0.02	*p* = 0.73	*p* < 0.01
Takahashi 2007	Hematologic Malignancy 88 AML	UCB	92	38	22 days	40 days	1.09	0.49	0.49	0.72	0.74
									
		MRD	71	40	17 days	22.5 days	1.0	1.0	1.0	1.0	1.0
				*p* = 0.83	*p* < 0.01	*p* < 0.01	*p* = 0.69	*p* = 0.01	*p* = 0.13	*p* = 0.26	*p* = 0.26
Gutman 2009	AML/ALL 53 AML						D100		2yr	2 yr	2 yr
		UCB	31	22	--	--	80.6%	--	20.6%	3.2%	76.2%
		MUD	31	25	--	--	67.7%	--	17%	25.8%	57.1%
							*p* = NS		*p* = 0.78	*p* = 0.018	*p* = 0.17
		MM URD	31	25	--	--	87.1%	--	29.2%	23%	47.8%
							*p* = NS		*p* = 0.41	*p* = 0.019	*p* = 0.041
Atsuta 2009	AML	UCB	173	38	--	--	32%	28%	30%	31%	36%
		URD	311	38	--	--	35%	32%	19%	24%	54%
							*p* = 0.39	*p* = 0.46	*p* = 0.004	*p* = 0.067	*p* < 0.001
**Myeloablative conditioning**											
Eapen 2010	AML/ALL 880 AML	UCB	165	28	24 days	52 days	1.0	1.0	1.0	1.0	1.0
		URD BM	332	39	19 days	28 days	0.78	0.63	1.69	0.85	1.15
		MM URD BM	140				0.59	0.59	1.06	0.84	0.93
		URD PB	632	33	14 days	19 days	0.57	0.38	1.62	0.85	1.12
		MM URD PB	256				0.49	0.46	0.95	0.91	0.91
				*p* < 0.0001	*p* < 0.0001	--	*p* = 0.001	*p* < 0.0001	*p* < 0.0001	*p* = 0.86	*p* = 0.09
Brunstein 2010	Hematologic Malignancy 476 acute leukemia	DUCB	128	25	26 days	53 days	1.0	1.0	1.0	1.0	1.0
		MRD	204	40	16 days	20 days	1.08	1.58	0.31	3.67	1.09
								*p* = 0.03	*p* < 0.01	*p* < 0.01	
		URD	152	31	19 days	21 days	1.83	1.71	0.61	3.05	0.85
							*p* < 0.01	*p* = 0.01		*p* < 0.01	
		MM URD	52	31	18.5 days	21 days	2.35	2.07	0.38	2.50	1.12
				*p* < 0.01	*p* < 0.01	*p* < 0.01	*p* < 0.01	*p* = 0.01	*p* < 0.01	*p* < 0.01	P = NS
**Myeloablative/Reduced intensity conditioning**											
Ponce 2011	Hematologic Malignancy 133 AML				MAC (RIC)	MAC (RIC)	D100	1 yr	D180	2 yr	2 yr
		DUCB	75	37	24 (10)	51 (38)	43%	28%	21%	20%	55%
		MRD	108	47	11 (11)	17 (12)	27%	31%	8%	19%	66%
									*p* = 0.017	9%	55%
		URD	184	48	11 (10)	18 (17)	39%	44%	13%		
				*p* = 0.071	*p* < 0.001	*p* < 0.001	*p* = 0.33	*p* = 0.044	*p* = 0.123	*p* = 0.813	*p* = 0.573
					(*p* = 0.084)	(*p* < 0.001)					
Raiola 2014	Hematologic, 232 acute leukemia 69% MAC					D50 (median)	D100	4 yr	D1000	4 yr	4 yr
		UCB	105	40 (18–64)	23 days	40 days	19%	23%	35%	30%	33%
		MRD	176	47 (15–69)	18 days	160 days	31%	29%	24%	40%	32%
		8/8 URD	43	42 (19–66)	17 days	100 days	21%	22%	33%	23%	36%
		7/8 URD	43	47 (17–62)	16 days	110 days	42%	19%	35%	30%	34%
		Haplo	92	45 (17–69)	18 days	118 days	14%	15%	18%	35%	43%
					*p* < 0.05	*p* < 0.01	*p* < 0.001	*p* = 0.053	*p* = 0.10	*p* = 0.89	*p* = 0.20
**Reduced intensity conditioning**											
Brunstein 2006	AML								1yr	2yr	2yr OS
		UCB	43	53	88%	--	51%	--	28%	35%	31%
				(22–68)							
		Sib PBSC	21	54	100%	--	62%	--	38%	35%	32%
				(19–69)	*p* = 0.1		*p* = 0.85		*p* = 0.43	*p* = 0.72	*p* = 0.62
Majhail 2008	Hematologic malignancies 29 AML				--	--	D100	3yr	D180		3yr
		UCB (88%	43	59			49%	17%	28%	--	34%
		DUCB)		(55–69)	--	--	42%	40%	23%	--	30%
		MRD	47	58			*p* = 0.20	*p* = 0.02	*p* = 0.23		*p* = 0.98
				(55–70)							
Majhail 2012	AML/MDS 70 AML						D100	2yr	2yr	2yr	2yr
		UCB (95%	60	61	--	--	45%	33%	25%	47%	22%
		DUCB)		(55–69)							
		MRD	38	63	--	--	38%	61%	25%	34%	34%
				(56–70)			*p* = 0.19	*p* = 0.04	*p* = 0.82	*p* = 0.19	*p* = 0.23
Brunstein 2012	AML/ALL						D100	2 yr	2 yr	2 yr	2 yr TF
	94% AML	DUCBT-TCF	121	55 (23–68)	1	--	1	1	1	1	1
	90% AML	8/8 PBCT	313	59 (23–69)	0.21	--	1.91	0.43	0.92	1.26	1.13
					*p* < 0.0001		*p* < 0.001	*p* < 0.001	*p* = 0.72	*p* = 0.155	*p* = 0.37
	82% AML	7/8 PBCT	111	58 (21–69)	0.21	--	1.44	0.45	0.57	1.15	0.88
					*p* = 0.013		*p* = 0.06	*p* < 0.001	*p* = 0.035	*p* = 0.495	*p* = 0.43
	75% AML	DUCBT-other	40	48 (21–67)	--	--	--	--	--	--	--
Chen 2012	Hematologic malignancies, 95 AML						D200	2yr	3 yr	3 yr	3 yr
		DUCBT	64	53 (19–67)	21.5	41	14.1%	21.9%	26.9%	42.7%	30%
		URD	221	58 (19–73)	13	19	20.3%	53.9%	10.4%	49.8%	40%
					*p* < 0.0001	*p* < 0.0001	*p* = 0.32	*p* < 0.0001	*p* = 0.0009	*p* = 0.09	*p* = 0.47
**Reduced intensity conditioning**											
Le Bourgeois 2013	Hematologic malignancies 38 AML							2yr	2yr	2yr	2yr
		DUCB	39	56	16	38	26%	26%	26.5%	23%	50.5%
				(22–69)							
		PBSC	52	59	17	0	31%	35%	6%	35.5%	59%
				(22–70)			P = NS	*p* = 0.02	*p* = 0.02	*p* = 0.32	*p* = 0.43
Weisdorf 2014	AML				D28	D90	D100	3 yr	3 yr	3 yr	3 yr
		UCB	205	59 (50–71)	69%	69%	35%	28%	35%	35%	28%
		8/8 URD	441	58 (50–75)	97%	91%	36%	53%	27%	35%	39%
					*p* < 0.0001	*p* < 0.0001	*p* = 0.69	*p* < 0.0001	*p* = 0.05	*p* = 0.95	*p* = 0.01
		7/8 URD	94	58 (50–72)	91%	89%	44%	59%	41%	26% at	34% at
					*p* < 0.0001	*p* < 0.0001	*p* = 0.14	*p* < 0.0001	*p* = 0.01	*p* = 0.13	*p* = 0.39
Malard 2015	AML				D42	>50K at D180					
		UCB	205	49 (19–69)	75%	56%	1	1	1	1	1
		10/10 URD	347	57 (19–70)	96%	84%	1.72	2.15	1.05	0.60	1.1
					*p* < 0.001	*p* < 0.001	*p* = 0.08	*p* = 0.08	*p* = 0.85	*p* = 0.02	*p* = 0.49
		9/10 URD	99	55 (19–68)	95%	75%	2.61	1.84	1.58	0.62	1.17
					*p* < 0.001	*p* < 0.001	*p* = 0.007	*p* = 0.23	*p* = 0.13	*p* = 0.07	*p* = 0.29

## 8. Haploidentical Transplantation

Allogenic HCT using a haploidentical (haplo) donor historically has been an attractive alternative approach given that donors are readily available for almost all patients. However, the initial experience with T-cell replete haplo-HCT was disappointing because of unacceptably high non-relapse mortality (NRM) and incidence of severe GVHD occurring in about half of patients [71,72,73]. In contrast, when *ex vivo* T-cell depletion platforms were utilized with the intention of minimizing GVHD, it led to an excessive increase in graft failure and infectious complication rates [74,75]. Novel strategies such as post-transplantation cyclophosphamide (PT-Cy), CD34+ “mega dose”, and α/β+ T–cell depletion have improved clinical outcomes and broadened the use haplo-HCT in recent years. The advantages of this alternative donor type include immediate donor availability, motivation of family donors, simplicity of use (at least in the context of PT-Cy), and low cost [76,77,78,79]. This is particularly important in developing countries where ease of access to international unrelated donors and UCB may be limited by cost and local policies. The main limitation of haplo-HCT is still a high risk of disease relapse, which in part can be addressed by the use of more intensive conditioning regimens. Ongoing studies are investigating post-transplantation maintenance therapy methods. Delayed immune reconstitution has also been a major limitation in some haplo-HCT platforms. Thus, several strategies were undertaken to minimize GVHD after haplo-HCT without significantly affecting immune reconstitution. Most of these strategies are still in the developmental stage, including those directed towards augmentation of immune reconstitution after T-cell-depleted haplo-HCT, such as infusion of pathogen-specific T-cells [80,81,82,83,84], suicide-gene expressing T-cells [85,86,87], regulatory T-cells (Tregs) [88,89,90], or *ex vivo* photodepletion of alloreactive donor T-cells [91,92]. Selective allodepletion in T-cell replete haplo-HCT was another strategy explored that includes *ex vivo* T-cell tolerance induction via co-stimulation blockade [93,94], *ex vivo* selective depletion of T-cells [95,96,97,98], and use of PT-Cy [9,76,99,100,101].

## 9. T-Cell Depleted Haploidentical Graft

Infusion of a mega-dose (>10 × 10^6^ cells/kg) of CD34+ selected cells is a strategy developed to overcome the poor hematopoietic engraftment of T-cell-depleted haploidentical grafts after myeloablative conditioning [8,102,103]. The Perugia group conducted a phase II trial of mega-dose infusion of CD34+ cells after intensive conditioning with Thio/Flu/TBI (8Gy) and rabbit ATG in 104 patients with acute leukemia [104]. Despite achievement of successful engraftment in over 90% of patients, the rate of NRM was still excessive (36.5%), mainly owing to infectious complications from delayed immune reconstitution. Similarly, EBMT reported unacceptable TRM (36%–61% ± 10% at two years) due to serious infections and poor immune reconstitution after T-cell-depleted myeloablative conditioning in 266 patients with acute leukemia [103]. However, this strategy led to a low incidence of GVHD and appeared promising. By using α/β/CD19+ T-cell depletion, Locatelli and colleagues recently reported that myeloablative haplo-HCT using Thio/Flu/TBI and ATG-based conditioning yielded acceptable engraftment, a low rate of GVHD, and faster immune reconstitution [98]. Although clinical outcomes of haplo-HCT appear to be improving with the use of these novel techniques, future studies will need to carefully weigh the cost and benefits of these approaches relative to other alternative donor choices.

## 10. Unmodified Haploidentical Graft with Post-Transplant Cyclophosphamide

The PT-Cy approach was pioneered by the John Hopkins group, and in recent years has become widely used at many transplant centers given its lower cost and ease of use. With this strategy, the bone marrow or peripheral blood stem cell graft is unmodified when it is infused into the patient, allowing alloreactive T-cells to proliferate until days +3 and +4 post-transplant. The patient then receives 50 mg/kg/day cyclophosphamide for *in vivo* T-cell depletion. As shown in animal models, and recently in humans, this dose of cyclophosphamide kills actively proliferating T-cells, but does not harm the hematopoietic progenitor and stem cells that are critical for blood count recovery and engraftment [78]. This strategy results in low risk of acute and chronic GVHD, which has been explained by the use of *in vivo* alloreactive T-cell depletion with high-dose Cy [78]. In addition, several recent studies suggest improvement of immune reconstitution with preserved memory T-cells when PT-Cy is used in T-cell-replete haplo-HCT [76,100]. Luznik and colleagues reported incidence rates of sustained engraftment, grades II-IV acute GVHD, and chronic GVHD of 87%, 34%, and <25%, respectively, among 67 RIC haplo-HCT recipients with hematological malignancies [105]. NRM at one year was acceptably low at 15%; however, the relapse rate was higher at 51%, resulting in two-year event-free survival (EFS) of only 26%. Similar results were observed in their most updated report of a phase II study involving 210 patients with hematological malignancies receiving RIC conditioning followed by bone marrow haplo-HCT and PT-Cy: the cumulative incidence rates of grade II-IV acute GVHD, chronic GVHD, five-year relapse, and EFS were 27%, 13%, 55%, and 27%, respectively [76]. Another recent study by Ciurea and colleagues compared the clinical outcomes of 65 haplo-HCT recipients with T-cell-replete haplo-HCT/PT-Cy *versus* T-cell-depleted peripheral blood HCT. They demonstrated the superiority of T-cell-replete haplo-HCT/PT-Cy in terms of one-year NRM (16% *vs.*42%, *p* = 0.03), chronic GVHD (8% *vs.* 18%, *p* = 0.03), PFS (45% *vs.* 21%, *p* = 0.03), and OS (66% *vs.* 30%, *p* = 0.02) [99]. Bashey and colleagues identified comparable rates of relapse, DFS, and OS between haplo-HCT/PT-Cy and MSD or adult URD HCT [9]. These results were reproduced by the Italian group in 459 allograft recipients of haplo-HCT/PT-Cy, MSD, matched URD, mismatched URD, and UCB [79]. Although in this study the UCB group had higher TRM and inferior survival compared to haplo-HCT, myeloablative conditioning with Thio/Bu/Flu or Bu/Cy was the most common regimen (83%) used for UCB allograft, which likely contributed to higher TRM and inferior survival in this group. A recent CIBMTR study examined the clinical outcomes of 2174 adults with AML receiving haplo-HCT/PT-Cy (*n* = 192) or matched URD (*n* = 1982) allograft and identified similar two-year survival rates for these two donor groups after both myeloablative conditioning and RIC [77]. The BMT-CTN conducted two parallel multicenter phase II trials of RIC haplo-HCT/PT-CY *versus* UCB HCT. Both of these alternative donor approaches produced comparable survival rates; however, a lower risk of TRM among recipients of RIC haplo-BMT/PT-CY was offset by a higher risk of relapse as compared to UCB HCT recipients [7]. A recent collaborative study by French and Italian groups retrospectively compared the clinical outcomes of haplo-HCT and UCB transplantation in 150 patients with various hematological malignancies [106]. While haplo-HCT in this study was mostly performed for a lymphoma diagnosis (84%), the UCB group in contrast was enriched with acute leukemia patients (63%) and those undergoing alloHCT with a significantly higher disease risk index (44% *vs.* 20%). These findings most likely contributed to a higher cumulative incidence of disease relapse and lower DFS after UCB transplantation as compared to haplo-HCT. However, these results had no impact on overall survival, and TRM was similar between the groups. A more recent and larger retrospective study by EBMT examined differences between haplo-HCT (32% PT-Cy-based) and UCB (49% Cy/Flu/TBI-based) allografts in 1446 patients with acute leukemia and identified delayed engraftment and a lower rate of chronic GVHD with UCB transplant, but otherwise similar long-term clinical outcomes with both donor types [107]. Newer strategies are being tested to further improve the outcomes of haplo-HCT/PT-Cy. Grosso and colleagues recently reported an encouraging two-year DFS of 74% among 30 patients with hematological malignancies who received myeloablative haplo-HCT with 1200 cGy TBI followed by infusion of fixed-dose donor T-cells, 48 hours later by high-dose Cy, and 24 hours later by selected donor CD34+ cell infusion [108]. In conclusion, clinical research to improve the outcomes of haplo-HCT has witnessed dramatic successes within the past decade (Table 2), allowing many adults with leukemia who do not have an available HLA-identical relative to receive allogenic HCT using readily available UCB or haploidentical donors.

**Table 2:** Haploidentical transplantation for acute leukemia in adults.

Reference	Malignancy	Conditioning Regimen	No of Patients	Median Age (range)	Median Time to ANC ≥500/µL	Median Time to Platelet >20 × 10^9^/L	aGVHD (II-IV) CI (%)	cGVHD CI (%)	TRM	Relapse	DFS
**T-cell depleted haplo-HCT**											
Aversa 2005	Acute leukemia 67 AML	Thio/Flu/TBI/ATG	104	33 (9–64)	11 days	15 days	8%	7%	36.5%	25% at 6mo	39% at median 22mo
Ciceri 2008	Hematologic	TBI-based:	173	37 (17–66)	12 days	--	5%	10%	36% CR1 54% CR2 66% advanced	16% CR1	48% CR1
	Malignancy	74% CR1/CR2		CR1/CR2						23% CR2	21% CR2
	173 AML	71% advanced		36 (16–63)						32%	1%
		Mostly with ATG		advanced						advanced	advanced
Chang 2009	Hematologic	Bu/Cy +ATG	133	15 (2–18)	12 days	15 days	--	--	--	--	--
	Malignancy										
	43 AML										
Chang BBMT 2009	Hematologic	Bu/Cy/cytarabine/ Semustine/rATG	348	24 (2–54)	13 days	16 days	--	--	--	--	--
	Malignancy										
	100 AML										
**Haplo-HCT with PT-Cy**											
Luznik 2008	Hematologic	NMA	68	46 (1–71)	15 days	24 days	34% at Day 200	5%–25% at 1-yr	15% at 1-yr	51% at 1-yr	26% at 2-yrs
	Malignancy	Flu/Cy/TBI									
	27 AML										
Kazamon 2010	Hematologic	NMA Flu/Cy/TBI	185	50 (1–71)	--	--	31%	15%	15% at 1-yr	--	35% at 1-yr
	Malignancy										
	49 AML										
Munchel 2011	Hematologic	NMA Flu/Cy/TBI	210	52 (1–73)	15 days	24 days	27%	13%	18% at 5-yr	55% at 5-yr	27% at 5-yr
	Malignancy										
	43 AML										
Solomon 2012	Hematologic	MA Flu/Bu/Cy	20	44 (25–56)	16 days	27 days	30%	35%	10% at 1-yr	40% at 1-yr	50% at 1-yr
	Malignancy										
	12 AML										
**Haplo-HCT with PT-Cy**											
Ciurea 2012	Hematologic Malignancy 42 AML/MDS	TCR-Haplo/PT-Cy:	65	45 (20–63)	18 days	26 days	20%	7%	16% at 1-yr	34% at 1-yr 36% at 1-yr	50% at 1-yr 21% at 1-yr
		26 MA & 6 NMA									
						
		TCD-Haplo/ATG: MA		36 (18–56)	13 days	12 days	11%	18%	42% at 1-yr		
Castagna 2014	Hematologic Malignancy 4 AML/MDS	NMA Flu/Cy/TBI	46 BM 23 PB	44 (19–68) 54 (25–65)	21 days 20 days	29 days 27 days	25% 33%	13% 13%	22% at 2-yr 12% at 2-yr	-- --	62% at 2-yr 62% at 2-yr
**Haplo-HCT with intensive immunosuppression**											
Huang 2006	Hematologic Malignancy 51 AML	MA	171	25 (2–56)	12 days	15 days	55%	47% at 2-yr	19%–31% at 2-yr	12%–39% at 2-yr	42%–68% at 2-yr
		Bu/Cy/ARA-C/									
		Semustine									
		IS: ATG/CSA/MTX/									
		MMF									
Huang 2009	Acute leukemia 108 AML	MA	250	25 (2–56)	12 days	15 days	46%	23% at 3-yr	19%–51% at 3-yr	12%–49% at 3-yr	25%–71% at 3-yr
		Bu/Cy/ARA-C/									
		Semustine									
		IS: ATG/CSA/MTX/									
		MMF									
Di Bartolomeo 2013	Hematologic Malignancy 45 AML	80%MA /20%RIC	80	37 (5–71)	21 days	28 days	24%	Extensive6% at 2-yr	36% at 1-yr	21% at 1-yr	38% at 3-yr
		Thio/Bu/Flu									
		IS: ATG/CSA/MTX/									
		MMF/Basiliximab									
**Haplo-HCT with intensive immunosuppression**											
Fu 2014	Hematologic Malignancy 34 AML	TBI/Cy/simustine/ATG Bu/Cy/simustine/ ARA-C/ATG	38 77	20 (13–46 24 (8–51))	13 days 12 days	19 days 16 days	32% 48%	61% at 1-yr 53% at 1-yr	13% at 1-yr 16% at 1-yr	27% at 2-yr 32% at 2-yr	58% at 2-yr 57% at 2-yr
**Comparative studies with Haplo-HCT**											
Lu 2006	Hematologic malignancies 69 AML	MA Haplo (Bu/Cy/ATG) MRD (Bu/Cy)	135 158				D100	2-yr	2-yr	2-yr	2-yr
				24	12 days	15 days	32%	55%	22%	18%	64%
				(3–50)							
				37	15 days	15 days	40%	56%	14%	13%	71%
				(5–50)	*p* < 0.001	*p* = NS	N = 0.13	*p* = 0.90	*p* = 0.10	*p* = 0.40	*p* = 0.27
Brunstein 2011	Hematologic malignancies 51 AML	RIC Haplo PT-Cy dUCB	50 50	48 (7–70) 58 (16–69)	16 days 15 days	24 days 38 days	D100 32% 40% *p* = 0.13	1-yr 13% 25%	1-yr	1-yr	1-yr
									7%	45%	48%
		
									4%	31%	46%
Bashey 2013	Hematologic malignancies 91 AML	50% MA Haplo PT-Cy MRD 8/8 URD	46 50 51	59 (50–71) 58 (50–75) 58 (50–72)	-- -- --	-- -- --	D180	2-yr	2-yr	2-yr	2-yr
							30%	38%	7%	33%	60%
							27%	54%	13%	34%	53%
							*p* = NS	*p* < 0.05	*p* = NS	*p* = NS	*p* = NS
							39%	54%	16%	34%	52%
							*p* = NS	*p* < 0.05	*p* = NS	*p* = NS	*p* = NS
**Comparative studies with Haplo-HCT**											
Raiola 2014	Hematologic malignancies 232 acute leukemia	69% MA UCB MRD 8/8 URD 7/8 URD Haplo	105 176 43 43 92	40 (18–64) 47 (15–69) 42 (19–66) 47 (17–62) 45 (17–69)		D50 (median)	D100	4 yr	D1000	4 yr	4 yr
					23 days	40 days	19%	23%	35%	30%	33%
					18 days	160 days	31%	29%	24%	40%	32%
					17 days	100 days	21%	22%	33%	23%	36%
					16 days	110 days	42%	19%	35%	30%	34%
					18 days	118 days	14%	15%	18%	35%	43%
					*p* < 0.05	*p* < 0.01	*p* < 0.001	*p* = 0.053	*p* = 0.10	*p* = 0.89	*p* = 0.20
Ciurea 2014 (ASH)	2174 AML	MA-Haplo PT-Cy MA-8/8 URD RIC-Haplo PT-Cy RIC-8/8 URD	104 1245 88 737	21–70	D30 CI	-- -- -- --	-- -- -- --	-- -- -- --	HR	HR	2-yr OS
					90%				1.0	1.0	47%
					97%				1.07	0.88	54%
					*p* = 0.01				*p* = 0.82	*p* = 0.40	*p* = 0.22
					93%				1.0	1.0	53%
					96%				2.35	0.76	49%
					*p* = 0.25				*p* = 0.03	*p* = 0.09	*p* = 0.25
Luo 2014	Hematologic malignancies 126 AML	TCR-Haplo MRD 8/8 URD	99 90 116	25 (9–55) 34 (16–56) 26 (10–50)	12 days 12 days 12 days	15 days 12 days 13 days	D90	2-yr		5-yr	5-yr
							42%	41%	31%	14%	58%
							16%	24%	5%	34%	64%
							*p* < 0.05	*p* =NS	*p* < 0.001	*p* = 0.008	*p* = NS
							40%	42%	22%	21%	58%
							*p* = NS	*p* = NS	*p* = NS	*p* = NS	*p* = NS
Ruggeri 2015	AML	Haplo (32% PT-Cy) UCB (49% Cy/Flu/TBI)	360 558			-- --		HR	HR	HR	HR
				44 (18–75)	23 days		27%	1.0	1.0	1.0	1.0
				45 (18–72)	17 days		31%	0.63	1.16	0.95	0.78
				*p* = 0.62	*p* < 0.01		*p* = 0.10	*p* = 0.008	*p* = 0.47	*p* = 0.76	*p* = 0.78

## 11. Unmodified Haploidentical Graft with Intensive Immune Suppression

Another approach to minimizing the rate of GVHD after haploidentical transplantation is the use intensive immunosuppression [109,110,111]. This strategy was first studied by Huang and colleagues, in which 250 patients with acute leukemia received a G-CSF-primed, unmanipulated, haploidentical, peripheral blood or bone marrow graft [111]. Myeloablative conditioning consisted of Bu/Cy/cytarabine/semustine and rabbit ATG, and the immunosuppression consisted of CSA, MMF, and methotrexate (MTX). Neutrophil engraftment was achieved in all except one patient, and the rates of grade II-IV acute GVHD, grade III-IV acute GVHD, and extensive chronic GVHD were 46%, 13%, and 23%, respectively. At three years, TRM and the relapse rate were higher for high-risk AML patients at 29% and 49%, respectively. In their most updated report Luo and colleagues compared their haplo-HCT experience with MRD and matched URD and identified higher TRM and lower relapse rate associated with haplo-HCT as compared to MRD graft; however, five-year LFS was similar in all three groups [112]. Most recently, the same group from China compared the TBI/Cy/simustine/ATG conditioning regimen with the Bu/Cy/simustine/cytarabine/ATG regimen in 115 patients with acute leukemia and reported similar clinical outcomes except for a higher rate of organ toxicities with the Bu-based regimen [113]. Another group from China reported their experience using T-cell-replete haplo-HCT with myeloablative conditioning consisting of Bu/Cy/cytarabine/lamustine and low-dose (10 mg/kg) ATG and immunosuppression consisting of CSA, MMF, and MTX [112]. They compared this haplo-HCT platform (*n* = 99) to MSD (*n* = 90) and URD (*n* = 116) allografts and identified comparable long-term DFS in all three groups. While there was no difference in other clinical outcomes between haplo-HCT and URD grafts, haplo-HCT recipients had a higher incidence of TRM and acute GVHD, but a lower relapse rate, than MRD recipients. In addition, a G-CSF-priming conditioning regimen in T-cell-replete haplo-HCT with intensive immunosuppression resulted in a lower relapse rate and superior LFS and OS as compared to non-G-CSF priming in a Southwest China multicenter randomized controlled study [114]. A similar strategy of intensive immunosuppression after T-cell-replete haplo-HCT has been evaluated in 80 patients with high-risk hematologic malignancies [115]. GVHD prophylaxis consisted of five drugs (ATG, CSA, MMF, MTX and basiliximab), and most patients (80%) received myeloablative conditioning consisting mainly of Thio/Bu/Flu. This therapeutic approach produced acceptable hematopoietic engraftment and low rates of acute and chronic GVHD, leading to a three-year LFS rate of 30% for high-risk patients. A calcineurin inhibitor-free, sirolimus-based immunosuppressive platform in combination with ATG, MMF, and rituximab was another modality that was used after trosulfan/Flu conditioning and T-cell-replete haplo-HCT; this approach demonstrated rapid T-cell immune reconstitution and promoted *in vivo* expansion of Tregs [116,117]. On the basis of these clinical investigations, it is reasonable to view T-cell-replete haplo-HCT in combination with intensive immunosuppression to be another promising haplo-HCT platform.

## 12. The Way Forward

Alternative donor transplantation is now a reality that allows almost every patient who requires alloHCT to proceed with this potentially curative treatment modality. Mismatched URD represents yet another alternative donor type, especially with our improved understanding of the HLA-system and HLA-matching, such as the identification of “permissive” mismatches [118]. Future advances in clinical care will require physicians to encourage their patients to participate in prospective clinical trials so that strategies to improve the clinical outcomes of HCT recipients using alternative donor types and platform options can be rigorously compared. One such clinical trial is the phase III, randomized, multicenter trial (BMT CTN protocol 1101) of RIC and double UCB HCT *versus* haplo-HCT with PT-Cy in adults with leukemia and lymphoma. This study will compare clinical outcomes as well as cost efficacy, quality of life, and immune reconstitution: important outcomes for defining the relative efficacy of the two donor types and helping to establish evidence-based standards of care.
